# Assisting Operators in Heavy Industrial Tasks: On the Design of an Optimized Cooperative Impedance Fuzzy-Controller With Embedded Safety Rules

**DOI:** 10.3389/frobt.2019.00075

**Published:** 2019-08-21

**Authors:** Loris Roveda, Shaghayegh Haghshenas, Marco Caimmi, Nicola Pedrocchi, Lorenzo Molinari Tosatti

**Affiliations:** ^1^Institute of Intelligent Industrial Technologies and Systems for Advanced Manufacturing of National Research Council, Milan, Italy; ^2^Istituto Dalle Molle di Studi sull'Intelligenza Artificiale, Scuola Universitaria Professionale della Svizzera Italiana, Università della Svizzera Italiana, Lugano, Switzerland

**Keywords:** human-robot cooperation, neural network human-robot interaction mapping, machine learning for autonomous control tuning, fuzzy logic safe controller, empowering humans, human-robot collaboration evaluation, variable impedance control

## Abstract

Human-robot cooperation is increasingly demanded in industrial applications. Many tasks require the robot to enhance the capabilities of humans. In this scenario, safety also plays an important role in avoiding any accident involving humans, robots, and the environment. With this aim, the paper proposes a cooperative fuzzy-impedance control with embedded safety rules to assist human operators in heavy industrial applications while manipulating unknown weight parts. The proposed methodology is composed by four main components: (i) an inner Cartesian impedance controller (to achieve the compliant robot behavior), (ii) an outer fuzzy controller (to provide the assistance to the human operator), (iii) embedded safety rules (to limit force/velocity during the human-robot interaction enhancing safety), and (iv) a neural network approach (to optimize the control parameters for the human-robot collaboration on the basis of the target indexes of assistance performance defined for this purpose). The main achieved result refers to the capability of the controller to deal with uncertain payloads while assisting and empowering the human operator, both embedding in the controller safety features at force and velocity levels and minimizing the proposed performance indexes. The effectiveness of the proposed approach is verified with a KUKA iiwa 14 R820 manipulator in an experimental procedure where human subjects evaluate the robot performance in a collaborative lifting task of a 10 kg part.

## 1. Introduction

Cooperative robotics applications are raising in industrial context due to the high potential of such robotic systems (Schmidtler et al., 2015; Donner and Buss, 2016; Agravante et al., 2019). Being characterized by hardware/software compliance and low inertia and having intrinsic safety features, such manipulators are extensively adopted in the working cells (Corrales et al., 2012). Despite such collaborative features, the need of safety regulation has become mandatory, developing standards to regulate safety during both non-physical and physical cooperation (ISO 20218, ISO/TS 15066). On top of such standards, many efforts have been made to apply such directives to the industrial field, considering the whole set of applications (Matthias et al., 2011; Michalos et al., 2015; Maeda et al., 2017).

### 1.1. Empowering Humans: State of the Art

Control methodologies to enhance physical human-robot cooperation in the industrial context are also required (Lasota et al., 2017) in order to relieve human operators from heavy loads and reduce/limit musculoskeletal disorders (European Week for Safety and Health at Work, 2017). Different approaches have been developed, considering programming-by-demonstration applications (Billard et al., 2008), improved human operator ergonomics in collaborative tasks (Shafti et al., 2018), human-robot mutual adaptation in collaborative tasks (Nikolaidis et al., 2017).

Empowering humans in industrial applications is one of most investigated area. Most of the proposed approaches relies on impedance (or admittance) control (Hogan, 1985), since it allows for the achievement of a tunable-soft robot behavior. While impedance control is high-performance in terms of human-robot interaction purposes, such methodology is affected by modeling errors (such as joint frictions), therefore requiring advanced dynamics compensation controllers (Tan et al., 2001; Lee et al., 2010; Roveda et al., 2017a).

On the basis of the state of the art, the following main features can be identified for classifying the control methodologies in the field: (i) variable impedance controllers (VIC), (ii) estimation of human arm dynamics parameters (EHADP), (iii) identification of human intention/motion (IHIM), (iv) capability to deal with uncertain/unknown payloads (UP), (v) human learning and reproduction of task skills (SR), and (vi) embedded safety rules (ESR). Table 1 shows the state of the art of the classification methods along with their main features.

**Table 1:** State of the art of classification methods along with implemented features.

**State of the art methods classification**						
**Method**	**VIC**	**EHADP**	**IHIM**	**UP**	**SR**	**ESR**
Tsumugiwa et al. (2002)	X	X				X
Duchaine and Gosselin (2007)	X		X			
Lichiardopol et al. (2009)			X	X		
Evrard et al. (2009)			X		X	
Xu et al. (2011)	X	X				
Jlassi et al. (2012)			X			X
Karayiannidis et al. (2013)			X			
Geravand et al. (2013)			X			X
Rozo et al. (2013)	X	X			X	
Peternel et al. (2014)	X	X	X		X	
Dimeas and Aspragathos (2014)	X		X			X
Li and Ge (2014)	X		X			
Ficuciello et al. (2014)	X					
Rozo et al. (2016)	X		X		X	
Noohi et al. (2016)			X			
Cherubini et al. (2016)			X			X
Mao et al. (2017)	X	X	X			
Gaz et al. (2018)			X			
Peternel et al. (2018)			X		X	
Proposed Method	X		X	X		X

In Tsumugiwa et al. (2002) the damping coefficient of the impedance control is adapted on the basis of the estimated human arm stiffness. The main contribution of the paper is the definition of a methodology for the variation of the damping coefficient of the impedance control in order to achieve a stable interaction with the human, making the human-robot cooperation safe. However, no force/velocity limitations to avoid dangerous situations are introduced in the controller. In Duchaine and Gosselin (2007) efforts have been made for the on-line tuning of the impedance control parameters based on human intentions, without the estimation of human arm dynamics. Human intentions of motion are estimated on the basis of the derivative of the interaction force. In Lichiardopol et al. (2009) a control strategy allowing the co-manipulation of loads with unknown and time-varying mass is proposed. The human applies force is estimated and a scaled version of such estimation is applied to the load to move the manipulated part. In Evrard et al. (2009) a cooperative lifting of a part is performed on the basis of the human motion characterization. The human motion is encapsulated in the collaborative robot behavior by a probabilistic model (a Gaussian Mixture Model is used to model the human behavior). In Xu et al. (2011) the use of a fuzzy logic approach (in order to deal with non-linear and uncertain systems while accounting for human like decision-making process) and of a neural network (in order to map and learn non-linear behavior) to tune a variable impedance controller for rehabilitation applications are investigated. The stiffness and damping parameters of the upper limb are estimated in order to provide the adaptation of the impedance control parameters. In Jlassi et al. (2012) the co-manipulation problem is addressed for handling tasks. Forces are treated only as exchanged physical signals, showing the robot how to move according to the willingness of the human operator. Indeed, this force gives the desired direction of displacement at every time step. Safety rules are included in such approach only limiting the robot velocity to a maximum value. In Karayiannidis et al. (2013) a method for estimating the constraints imposed by a human agent on a jointly manipulated object is proposed. These estimates can be used to infer knowledge of where the human is grasping an object, enabling the robot to plan trajectories for manipulating the object while subjected to the constraints. In Geravand et al. (2013) a collision detection algorithm is developed in order to distinguish from desired and undesired contacts between human and robot. In such a way, reaction behaviors are applied to the robot in order to react to the contact. In Rozo et al. (2013) human behavior in manipulation tasks are learned (in particular with respect to impedance parameters). Such behaviors are applied to a robot performing a co-manipulation task in collaboration with the human. In Peternel et al. (2014) an approach to efficiently teach robots how to perform dynamic manipulation tasks in cooperation with a human partner is proposed. The approach utilizes human sensorimotor learning ability where the human tutor controls the robot through a multi-modal interface to make it perform the desired task. During the tutoring, the robot simultaneously learns the action policy of the tutor and through time gains full autonomy. In Dimeas and Aspragathos (2014) a fuzzy inference system is designed that relies on the measured velocity and the force applied by the operator to modify on-line the damping of the robot admittance, based on expert knowledge for intuitive cooperation. Safety rules are applied only to the damping parameter of the impedance control in order to avoid low values and, therefore, instabilities in the collaboration. No limitations on forces or velocities are applied. In Li and Ge (2014) an adaptive impedance control is proposed for a robot collaborating with a human partner in the presence of unknown motion intention of the human partner and unknown robot dynamics. Human motion intention is on-line estimated, making use of neural networks. Such estimation is used in order to tune the adaptive impedance controller. In Ficuciello et al. (2014) the problem of controlling a redundant robot arm executing a cooperative task with a human who guides the robot through direct physical interaction is addressed. The problem is tackled by allowing the robot end-effector to be compliant according to an impedance control law defined in the Cartesian space. The proposed idea relies on the use of the robot redundancy to ensure a decoupled apparent inertia at the robot end-effector enabling a more flexible choice of the impedance parameters thus improving the performance during manual guidance. In Rozo et al. (2016) a framework for a user to teach to the robot collaborative skills from demonstrations is designed. Specifically, an approach that combines probabilistic learning, dynamical systems, and stiffness estimation to encode the robot behavior along the task is proposed. In Noohi et al. (2016) a model that allows for the computation of the interaction force during a dyadic cooperative object manipulation task is proposed. Such model is used to enhance the human-robot cooperation. In Cherubini et al. (2016) an approach enhancing physical human-robot interaction is proposed. In particular, safety rules are applied in order to limit the interaction force and to adapt the robot behavior on the basis of the recognition of the human hand (through image processing). In Mao et al. (2017) a dynamic fuzzy variable impedance control algorithm is proposed for human-robot cooperation. In order to estimate the intention of human for co-manipulation, a fuzzy inference system is set up to adjust the impedance parameter. Aiming at regulating the output fuzzy universe based on the human arm stiffness, an on-line stiffness identification method is developed. In Gaz et al. (2018) a collaborative polishing task is considered. The human applied interaction forces and the polishing forces are identified. The human applied interaction forces are then used in order to enhance the human-robot collaboration. In Peternel et al. (2018) a method for human-robot collaboration, where the robot physical behavior is adapted on-line to the human motor fatigue, is proposed. As the collaborative task is performed under the human lead, the robot learns gradually the parameters and trajectories related to the task execution. In the meantime, the robot monitors the human fatigue during the task performance or execution.

Despite the number of contributions in the field of empowering humans, as a result of the state of the art analysis, the approaches involving the manipulation of (partially) unknown payloads and the definition of safety rules are very limited. Moreover, in most of the cases, the safety rules guarantee only the stability of the system (i.e., without any limitation of the resulting force/velocity). Even if the manipulator behavior is proven to be stable, extra assistance (resulting in extra velocity of the robot or extra force applied to the human) may occur, resulting in non-ergonomic human-robot cooperation or even in a dangerous interaction. Moreover, considering the dynamic and no a-priori defined working scenario (e.g., manipulating unknown objects), the payload manipulated by the human and by the robot can be unknown. Such practical situations cannot be faced by the above described approaches, which require a perfect tuning of the manipulated payload.

### 1.2. Paper Contribution

In order to overcome the above described issues (i.e., provide safety features while providing assistance during the co-manipulation of unknown weight objects), the paper proposes a cooperative fuzzy-impedance control with embedded safety rules to assist human operators in heavy industrial applications. The proposed methodology is composed by four main components: (i) an inner Cartesian impedance controller, (ii) an outer fuzzy controller, (iii) embedded safety rules, and (iv) a neural network approach. (i) the inner Cartesian impedance controller allows to achieve the robot compliant behavior. (ii) the outer fuzzy controller allows to calculate the assistance to the human operator. Three input membership functions have been defined on the basis of the interaction force, the derivative of the interaction force and the end-effector velocity. On the basis of such membership functions it is possible to identify the human intentions of motion. The fuzzy control calculates the assistance to be provided to the human operator on the basis of the defined output membership function. The impedance control set-point is therefore updated compensating for the unknown manipulated component weight (both in motion—relieving the human from the load—and in stop conditions—avoiding any drift of the robot position due to the constant force applied by the manipulated weight). (iii) the embedded safety rules ensure a safe robot behavior (both limiting robot velocity and interaction forces). In particular, embedded safety rules consist in the definition of four membership functions sets for the fuzzy controller. Each set is related to a specific velocity range (defined based on ISO 10218-1:2011). The impedance control set-point is calculated by (ii) in order to satisfy the limitations defined by the embedded safety rules. (iv) the neural network approach allows to optimize the assistance levels of the output membership function of the fuzzy controller (i.e., the gains of the control law) together with the impedance control parameters (i.e., the stiffness and damping parameters) in order to improve the human-robot cooperation on the basis of human effort and trajectory smoothness indexes. Effort indexes have been defined in order to monitor the physical stress of the human operator while cooperating with the manipulator. Two indexes have been proposed: (i) exploiting interaction force measurements, an effort index has been defined in order to monitor the interaction between the human and the robot; (ii) exploiting electromyography signals measurements (EMGs), an index related to the muscular activity of the human arm has been defined in order to monitor the human stress. Trajectory smoothness index has been defined in order to monitor the naturalness of the human-robot interaction. Exploiting the robot position measurements, a jerk-related index has been proposed. Experiments have been performed by a single subject to gather data for the training of the Neural Network and then to perform the optimization of the control parameters. The impedance stiffness and damping parameters and the assistance levels have been varied through the experiments. The manipulated payload mass has been also varied during the training experiments (using 0, 5, and 10 kg payloads) in order to map the control parameters variation with respect to this variable.

The effectiveness of the proposed approach has been verified with a KUKA iiwa 14 R820 manipulator in an experimental procedure where human subjects evaluated the robot performance in a collaborative lifting task of a 10 kg part (weight unknown to the robot controller). The results have shown that even if the control parameters have been tuned on a single operator, the controller is still optimized for multiple operators. Qualitative and quantitative analysis have been performed on such experimental results in order to better highlight the capabilities of the proposed approach.

The proposed controller is therefore capable to assist the human operator while manipulating an unknown component weight, relieving him/her from the lifted load and ensuring safety (at force and velocity levels). It has to be note that the weight of the manipulated part is not known by the robot controller. In fact, only a rough estimation of the part weight is required to initially set the controller parameters. However, considering the results obtained from the optimization procedure, the optimized control parameters are not varying much with the different applied payloads. Therefore, even in the presence of uncertainties in the payload estimation, the selected control parameters can be considered sub-optimal for the human-robot cooperation task. As shown in Table 1, such method includes the most of the capabilities of the available state of the art methods, allowing to empower the human even without any estimation of the arm dynamics (that may affect the controller reducing the performance in occurrence of estimation errors) and ensuring safety (both at force and velocity level).

## 2. Problem Formulation

In this section the challenges faced by the paper in the human-robot cooperation are analyzed. The approaches proposed by the paper to solve them are also proposed.

- Implementation of a **compliant and tunable soft behavior** for the controlled manipulator: such a control feature allows to perform a natural interaction between the human and the robot. An inner control loop defined by a Cartesian impedance controller is proposed by this paper to achieve such goal.
- **Empower the human operator**, assisting him/her during the execution of the cooperative application while relieving from the unknown manipulated load: such a control feature allows to improve the working conditions of the human operator. An outer control loop defined by a fuzzy controller is proposed by this paper to achieve such goal.
- **Ensure safety** in human-robot collaboration. Safety rules (at force and velocity level) embedded in the outer fuzzy controller are proposed in this paper. The embedded safety rules define four sets of input membership functions of the fuzzy controller (based on four velocity ranges defined on the basis of ISO 10218-1:2011) to be selected on the basis of the velocity state of the manipulator while cooperating with the human.
- **Optimize the human-robot cooperation**: improve the task ergonomics and the naturalness of the interaction. A Neural Network approach is proposed in this paper in order to optimize the control parameters. An off-line mapping of the control parameters (both inner impedance controller parameters—i.e., stiffness and damping—and assistance levels of the fuzzy controller) with respect to the manipulated payload and to the proposed performance indexes (i.e., effort based indexes and trajectory smoothness index described in section 3.4.1) is performed in order to optimize the human-robot interaction.

**Figure 1:**
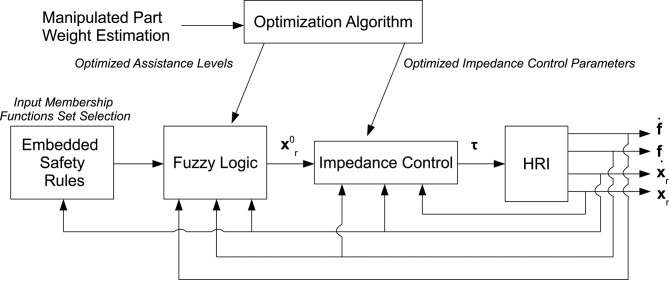
Control scheme showing the inner control loop (i.e., the Cartesian impedance control, calculating the torque vector τ to be given to the robot, achieving the compliant robot behavior), the outer control loop (i.e., the fuzzy controller, calculating the impedance control set-point ${\text{x}}_{r}^{0}$ providing the assistance to the human operator and relieving him/her from the manipulated load), the embedded safety rules (i.e., the set of input membership functions, ensuring safety during the human-robot cooperation), the optimization procedure (i.e., the neural network approach, defining the control parameters optimizing the human-robot cooperation), and the feedbacks.

Figure 1 shows the complete control scheme, including the inner control loop (i.e., the Cartesian impedance controller), the outer control loop (i.e., the fuzzy controller), the embedded safety rules and the off-line optimization of the control parameters.

## 3. Methodology

In this section the cooperative fuzzy-impedance control with embedded safety rules is described. In particular, the four main components of the proposed method (i.e., i) the inner Cartesian impedance controller, (ii) the outer fuzzy controller, (iii) the embedded safety rules, and (iv) the neural network approach for control parameters optimization) are described separately to highlight each specific contribution.

### 3.1. Inner Control Loop: Cartesian Impedance Control

The inner Cartesian impedance controller allows for the achievement of the compliant robot behavior while cooperating with the human operating and/or interacting with the surrounding environment. Such compliant behavior allows for the implementation of an intrinsic safe behavior of the robot system. Moreover, the Cartesian impedance control defines the foundation for the development of the outer controller. The inner Cartesian impedance control results in the following dynamics equations (Siciliano and Villani, 2000):

(1) $$\begin{array}{l} {\text{M}}_{r}{\ddot{\text{x}}}_{r}+{\text{D}}_{r}{\dot{\text{x}}}_{r}+{\text{K}}_{r}\Delta {\text{x}}_{r}={\text{f}}_{r} \end{array}$$

where **M**_*r*_, **D**_*r*_, **K**_*r*_ are the impedance matrices composed by both the translational and rotational parts, $\Delta {\text{x}}_{r}={\text{x}}_{r}-{\text{x}}_{r}^{0}$ (where ${\text{x}}_{r}^{0}$ is the six DoFs reference for the impedance controller and **x**_*r*_ is the robot Cartesian measured position), ${\dot{\text{x}}}_{r}$ is the robot Cartesian velocity, ${\ddot{\text{x}}}_{r}$ is the robot Cartesian acceleration and **f**_*r*_ is the measured wrench (including forces and torques at the end-effector).

The KUKA iiwa 14 R820 enables a task space visco-elastic behavior (as for the previous KUKA LWR 4+ manipulator Albu-Schäffer et al., 2007), with diagonal positive mass matrix (as shown in Roveda, 2015), and decoupled diagonal tunable stiffness and damping matrices **K**_*r*_: = *diag*(*K*_*r, x*_, *K*_*r, y*_, *K*_*r, z*_, *K*_*r*,_φ__*x*__, *K*_*r*,_φ__*y*__, *K*_*r*,_φ__*z*__), **D**_*r*_ : = *diag*(*D*_*r, x*_, *D*_*r, y*_, *D*_*r, z*_, *D*_*r*,_φ__*x*__, *D*_*r*,_φ__*y*__, *D*_*r*,_φ__*z*__), where ${\text{D}}_{r}=2M_{r}\sqrt{{\text{K}}_{r}{\text{M}}_{r}^{-1}}{\text{h}}_{r}$ and **h**_*r*_ is the damping ratio. In such a way, a compliant behavior can be set for the robot manipulator enabling the human-robot interaction.

### 3.2. Outer Control Loop: Fuzzy Impedance Controller

While the inner controller allows for the definition of a specific compliant behavior of the manipulator, the outer controller allows for the calculation of the set-point of the inner controller in order to empower and assist the human operator while relieving him/her from the unknown manipulated weight. The proposed outer controller is a fuzzy controller with three input membership functions defined on the basis of the interaction force, the derivative of the interaction force and the robot velocity, and with one output membership function allowing for the calculation of the assistance level to be given to the human operator on the basis of the cooperation state. The rules defined for the fuzzy impedance controller allows to recognize the human intention of motion in order to assist him/her in the desired direction of motion.

#### 3.2.1. Control Design

**Figure 2:**
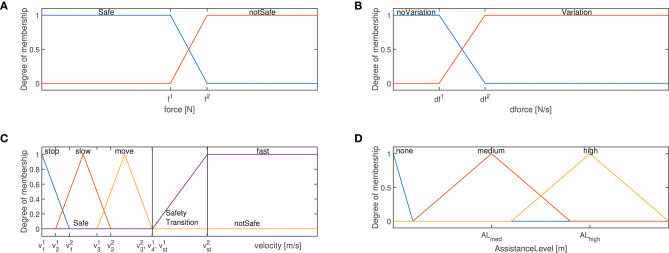
**(A)** Force membership functions: *safe*, *notsafe* states are highlighted. **(B)** Force derivative membership functions: *noVariation*, *Variation* states are highlighted. **(C)** Velocity membership functions: *stop*, *slow*, *move*, *fast* states are highlighted. Moreover, the safe and unsafe zones are highlighted. **(D)** Assistance level membership functions: *none*, *medium*, *high* states are highlighted.

The outer controller calculates the set-point of the inner controller in order to empower the human operator while relieving him/her from the unknown manipulated weight. To satisfy these goals, a fuzzy control is proposed with three input membership functions:

- (i) input membership function based on the interaction force **f**_*r*_ (Figure 2A): it allows for the definition of a safety feature to limit the assistance to the human operator on the basis of the measured interaction force. In particular, if the interaction force overcomes the defined limit, the assistance to the human operator is terminated in order to avoid any extra assistance that may result in extra motor torques;
- (ii) input membership function based on the derivative of the interaction force ${\dot{\text{f}}}_{r}$ (Figure 2B): it allows for the identification of the intention of motion of the human. In particular, on the basis of the variation of the interaction force it is possible to predict the intention of motion of the human as in Duchaine and Gosselin (2007);
- (iii) input membership function based on the measured robot Cartesian velocities ${\dot{\text{x}}}_{r}$ (Figure 2C): it allows for both the definition of a safety feature to limit the assistance to the human operator on the basis of the robot velocity and the identification of the intention of motion of the human. In particular, if the velocity overcomes the defined limit $v_{4}^{1}=v_{st}^{1}$, the assistance to the human operator is decreased with the increase of the robot velocity until reaching a zero assistance for the maximum allowed robot velocity $v_{st}^{2}$. In such a way, any extra velocity of the robot that may result in dangerous situations is avoided. Such membership function, in fact, defines three operating zones: a *Safe* operation zone, a *Safety Transition* operation zone and a *notSafe* operation zone. In addition, four ranges of velocity have been defined to monitor the robot motion, recognizing the intentions of motion of the human (considering the *stop* state—i.e., a state in which the robot is not moving—, the *slow* state—i.e., a state in which the robot is moving slowly—, the *move* state—i.e., a state in which the robot is moving with an affordable velocity—, and the *fast* state—i.e., a state in which the robot is moving too fast).

The definition of each membership function is shown in the Figure 2, identifying the different application phases for the human-robot collaboration. In particular, as shown in Figure 2A, two states have been defined for (i): *Safe* and *notSafe* (defined by the variables *f*^1^ and *f*^2^). While in the first state the human is applying a force allowing a safe-human robot interaction, in the second state the applied force is excessive and could result in dangerous interaction. The *notSafe* state defines therefore a dangerous zone in which the assistance to the human operator is no longer provided. As shown in Figure 2B, two states have been defined for (ii): *noVariation* and *Variation* (defined by the variables *df*^1^ and *df*^2^). While in the first state no human intention to move the robot is identified, in the second state the subject aims to move the robot (as verified by the change in the interaction force). As shown in Figure 2C, four states have been defined for (iii): *stop, slow, move* and *fast* (defined by the variables $v_{1}^{1}$, $v_{1}^{2}$, $v_{2}^{1}$, $v_{2}^{2}$, $v_{3}^{1}$, $v_{3}^{2}$, $v_{4}^{1}$, $v_{4}^{2}$, $v_{st}^{1}$, $v_{st}^{2}$). While in the first state the robot is not moving, in the second state it moves slowly, in third one it moves with a reasonable velocity and in the last one it is moving dangerously fast. The *fast* state defines therefore a dangerous zone in which the assistance to the human operator is no longer provided.

One output membership function is defined, in order to on-line calculate the assistance level **AL** to be given to the human operator, deforming the impedance control set- point:

(2) $$\begin{array}{l} {\text{x}}_{r}^{0}\left(t+1\right)={\text{x}}_{r}\left(t\right)+\text{A}\text{L}\left(t\right)sign\left({\dot{\text{f}}}_{r}\left(t\right)\right) \end{array}$$

where the impedance set-point is updated at time *t* + 1 on the basis of the control gains and feedbacks at time *t*.

The diagonal matrix of the level of assistance **AL** deforms the impedance control set-point to empower the capabilities of the operator during the execution of the collaborative task, relieving him/her from the unknown manipulated load. The corresponding output membership function is defined as in Figure 2D, where three levels of assistance have been defined: *none, medium*, and *high* (defined by the variables *AL*_*med*_ and *AL*_*high*_). While the first state indicates that no assistance has to be provided to the operator (due to no-motion requirement or due to safety violation—see the rules defined below), the second and third states define two different assistance levels for empowering the human operator on the basis of the current collaboration state.

The following rules have been implemented to calculate the level of assistance (**AL**) from the inputs (i.e., force **f**_*r*_, derivate of the force ${\dot{\text{f}}}_{r}$ and robot velocity ${\dot{\text{x}}}_{r}$) on the basis of the defined states of the input membership functions:

(3) $$\left\{ \begin{array}{l} \#1\;If{\dot{x}}_{r}:stop\;\&\&\;{\dot{\;f}}_{r}:noVar\&\&\;f_{r}:safe\;then\;AL:none\\ \#2\;If{\dot{x}}_{r}:stop\;\&\&{\dot{\;f}}_{r}:Var\&\&\;f_{r}:safe\;then\;AL:med\\ \#3\;If{\dot{x}}_{r}:slow\;\&\&{\dot{\;f}}_{r}:noVar\&\&\;f_{r}:safe\;then\;AL:med\\ \#4\;If{\dot{x}}_{r}:slow\;\&\&{\dot{\;f}}_{r}:Var\&\&\;f_{r}:safe\;then\;AL:high\\ \#5\;If{\dot{x}}_{r}:move\;\&\&\;{\dot{f}}_{r}:noVar\&\&\;f_{r}:safe\;then\;AL:med\\ \#6\;If{\dot{x}}_{r}:move\;\&\&{\dot{\;f}}_{r}:Var\&\&\;f_{r}:safe\;then\;AL:high\\ \#7\;If{\dot{x}}_{r}:fast\;then\;AL:none\\ \#8\;If\;f_{r}:notsafe\;then\;AL:none \end{array}\right.$$

where *noVar = noVariation, Var = Variation, med = medium*.

More in details, rule #1 aims at imposing no assistance when both no-force variation and no motion of the robot is observed. This means that the operator does not want to move the manipulator. Since the mass of the manipulated load is not well-known to the robot controller, such rule allows compensating for the uncertainties, avoiding any drift of the robot position due to the constant gravity force applied by the payload. Rule #2 aims at imposing a medium level of assistance when no motion of the robot is observed but a variation of the force is measured. This refers to the situation when the operator wants to start the cooperation. Rules #3 and #5 aim at imposing a medium level of assistance. This means that the operator would like to continue the robot motion with the current robot velocity. Rules #4 and #6 aim at imposing a high level of assistance. In both the cases, the operator would like to modify the robot motion. Considering rules from #2 to #6, the robot assists the operator empowering him/her while compensating for the weight of the manipulated part. The force input state is, in fact, *safe*, therefore enabling the safe cooperative controller to assist the operator. Rule #7 keeps the operator in a safe range of velocity, disabling the assistance to the operator thus avoiding an extra velocity of the manipulator. A safety transition zone is defined in which the assistance to the human operator decreases with the increasing of the robot velocity, until reaching a zero assistance for the maximum allowed robot velocity $v_{st}^{2}$. Rule #8 disables any level of assistance when the operator is imposing a high force. In this case, in fact, the risk is related to an excessive interaction that may result in unsafe situations.

**Remark 1**. On the basis of the target application (i.e., payload, specific human-robot interaction task, robot, etc.) and on the basis of the ISO 20218 it is possible to tune the parameters defining the membership functions of the fuzzy controller. For the evaluation of the proposed method, the values implemented for the specific validation scenario are detailed in section 4.1. The proposed method is general and applicable to any robotic system or application.

**Remark 2**. The developed control scheme does not require the dynamic model of the human operator arm. Based on the interaction force **f**_*r*_, the derivative of the interaction force ${\dot{\text{f}}}_{r}$, and the velocity ${\dot{\text{x}}}_{r}$ signals describing the operator intention are used by the algorithm to calculate the target level of assistance (separately for each degree of freedom—DoFs—since the implementation of the inner Cartesian impedance controller) during the task execution.

**Remark 3**. It has to be note that the defined states of the membership functions are managed by the fuzzy controller. In such a way, the algorithm is capable to evaluate the complex rules defined above, being capable to identify the resulting rule to be applied (i.e., by *defuzzifying* the result of the defined rules). Moreover, by defining the inference method to be applied to calculate the output of the fuzzy controller (Zimmermann, 1996), it is possible to calculate the result of the applied rules in the continuous space of solutions, avoiding any discretization of the results. In the proposed paper, the *max-min* inference method has been applied.

**Remark 4**. It has to be note that the interaction force cannot be used to identify the intentions of motion of the human. In fact, since the manipulated payload is supposed to be (partially) unknown (also without any knowledge of the center of mass of the payload), it is not possible to correlate directly the intentions of motion of the human to the measured interaction force **f**_*r*_ only.

**Remark 5**. Considering the safety rules in (3), their application is affected by the applied control parameters (i.e., impedance parameters and assistance levels). Given the highly non-linear system composed by the human and the robot in interaction with an unknown payload mass, it is not possible to define the control parameters with an analytical procedure. Therefore, an optimization of the control parameters is required to optimize the control performance on the basis of specified indexes. Such an optimization procedure is described in section 3.4.

**Remark 6**. ${\dot{\text{f}}}_{r}$ is filtered at 20 Hz because force measurements contain noise. It has to be underline that the noise may result in decreasing performance of the proposed controller. However, since the low-bandwidth of the target application (for impedance control-based method, 2 Hz is sufficient for the controller bandwidth; Hogan, 1985) such issue can be avoided by properly filtering the force signals.

**Remark 7**. The update of the impedance control set-point is performed at 200 Hz.

### 3.3. Embedded Safety Rules

In order to shape and adapt the assistance to the human operator ensuring safety in all the states of the collaboration, four robot velocity ranges have been defined. In fact, as the robot velocity increases the assistance to the human has to be bounded, in order to avoid any unexpected unsafe interaction. Considering high payloads in co-manipulation, such an issue becomes more critical, since any unexpected interaction may result in a dangerous situations. Therefore, to avoid any uncontrolled velocity and interaction force for safe interaction, the faster the robot is, the more limited is the assistance to the human.

On the basis of the four velocity ranges, four sets of the force and derivative of the force input membership functions have been defined. Exploiting the adaptation of the input membership functions and on the basis of the proposed fuzzy control rules, the output membership function calculates on-line the assistance level to be given to the human operator, ensuring safety.

The four velocity ranges have been defined accordingly to the membership function in Figure 2:

(4) $$\{\begin{array}{ll} \; & range\;\#1:v_{1}^{1}-v_{1}^{2}\\ \; & range\;\#2:v_{2}^{1}-v_{2}^{2}\\ \; & range\;\#3:v_{3}^{1}-v_{3}^{2}\\ \; & range\;\#4:v_{4}^{1}-v_{3}^{2} \end{array}$$

where $v_{i}^{1}$ indicates the lower bound for the velocity range *#i* and $v_{i}^{2}$ indicates the upper bound for the velocity range *#i*. In section 4.1.1 the velocity ranges are defined for the proposed evaluation scenario in section 5.

Considering that the faster the robot moves, the more dangerous would be for the operator, the manipulator velocity is the most suitable parameter for selecting the set of input membership functions, i.e., it is the dominant variable in the calculation of the safety rules. Consequently, the input membership functions related to the interaction force **f**_*r*_ (Figure 2A) and to the derivative of the interaction force ${\dot{\text{f}}}_{r}$ (Figure 2B) are shaped for each velocity range to limit the assistance level given by the robot to the human operator in order to ensure safety. More in details, as the velocity increases, the *Safe* zone related to the force input membership function and the *noVariation* zone related to the derivative of the force input membership function decrease. On the basis of the above defined rules for the calculation of the assistance level, this results in a limitation of the assistance as soon as the velocity of the robot increases. Therefore, the force and derivative of the force input membership functions parameters have been defined as follows on the basis of the velocity range defined in 4 (see section 4.1.2 for the implementation of the following parameters values):

(5) $$\{\begin{array}{ll} range & \#1:f_{1}^{1},f_{1}^{2}df_{1}^{1},\;df_{1}^{2}\\ range & \#2:f_{2}^{1},f_{2}^{2}\;df_{2}^{1},df_{2}^{2};\\ \; & with\;f_{2}^{1}>f_{1}^{1},f_{2}^{2}>f_{1}^{2},df_{2}^{1}>df_{1}^{1},df_{2}^{2}>df_{1}^{2}\\ range & \#3:f_{3}^{1},f_{3}^{2}df_{3}^{1},df_{3}^{2};\\ \; & with\;f_{3}^{1}>f_{2}^{1},f_{3}^{2}>f_{2}^{2},df_{3}^{1}>df_{2}^{1},df_{3}^{2}>df_{2}^{2}\\ range & \#4:f_{4}^{1},f_{4}^{2}\;df_{4}^{1},df_{4}^{2};\\ \; & with\;f_{4}^{1}>f_{3}^{1},f_{4}^{2}>f_{3}^{2},df_{4}^{1}>df_{3}^{1},df_{4}^{2}>df_{3}^{2} \end{array}$$

### 3.4. Neural Network Based Optimization Algorithm

Since the performance of the proposed controller is affected by both the assistance level (i.e., the *medium* *AL*_*med*_ and *high* *AL*_*high*_ values shown in Figure 2D) and the impedance control parameters (i.e., stiffness and damping parameters) and it is not possible to set analytically such values (due to the complex interaction between the human and the robot), an optimization of such a set of parameters has been done via a Neural Network (NN) approach (Marquardt, 1963). The algorithm aims at minimizing the defined performance indexes in section 3.4.1 to improve the human-robot cooperation (in terms of human effort or smoothness of the interaction, see section 3.4.1). The algorithm comes from the use of Neural Networks as a black-box model to fit a set of input/output data (defined in section 4), mapping the human-robot cooperation performance (on the basis of the defined performance indexes in section 3.4.1). Impedance control parameters (i.e., stiffness and damping parameters) and the output membership-functions assistance levels *AL*_*med*_, *AL*_*high*_ are considered as the inputs of the Neural Network. Such parameters have to be optimized to enhance the human-robot cooperation. In addition, the weight of the manipulated component *M*_*o*_ must be considered as an input of the Neural Network, in order to map any possible variation of the optimized control parameters with respect to such variable. By contrast, the optimization indexes represent the output quantities of the Neural Network. The aim of the proposed optimization approach is, therefore, to identify the control-parameters set optimizing the cooperation between human and robot while co-manipulating a target component. In the following, the optimization criteria are described.

#### 3.4.1. Performance Indexes Definition for the Optimization Process

In order to optimize the safety-based empowering of the human to perform heavy tasks, three different criteria to evaluate the performance of the cooperation between the human and the robot have been selected: (i) maximum movement smoothness, (ii) minimum effort based on the measured interaction force, and (iii) minimum muscular activity.

1. **Smoothness index iNJ**: it is known that smoothness is an indirect measure of the human motor control capacity being used in medicine to evaluate patients' levels of impairment and the efficacy of therapies (Caimmi et al., 2008). In fact, upper-limb natural movements are highly smooth and show high repeatability when performed cyclically (Caimmi et al., 2015). There are different methods to measure smoothness, all based on the elaboration of the position jerk (i.e., the third derivative of the position). The average Normalized Jerk (*NJ*) has been chosen for this study because, being dimensionless, can be used to compare movements of different lengths and execution times (Caimmi et al., 2015). The first index is therefore related to the normalized jerk, and it has been calculated as follows:
  (6) $$iNJ(j)=\sqrt{1/2\sum_{i=1}^{n}(Jerk(j){)}^{2}\Delta t{\left(T_{END}-T_{START}\right)}^{5}L^{2})}$$
  where *Jerk* is the third derivative of the robot position ($Jerk={\dddot{x}}_{r}$), *T*_*END*_ − *T*_*START*_ the movement duration, *L* is the distance between the start and end positions and Δ*t* is the sampling time. The index is calculated for each jth degree of freedom, separately.
2. **Force index iF**: the second index defines a measure of the effort required by the human to cooperate with the manipulator on the basis of the interaction force **f**_*r*_ measurement. The idea behind this choice is that the better the robot is assisting the human, the lower are the forces required during the cooperation. In order to compare movements of different lengths and execution times, the root mean square (RMS) of the force has been used as the proposed index, dividing it by Δ*x*_*r,z*_, the difference in height between the start and end points:
  (7) $$iF(j)=\frac{\sqrt{\sum_{i=1}^{n}{\left(f_{r}(j)\right)}^{2}/n)}}{\Delta x_{r,z}}$$
  where *n* is the total number of samples. The index is calculated for each jth degree of freedom, separately.
3. **Muscular activity index iEMG**: the third index is a measure of the operator effort based on the level of muscular activity measured during task performance. Once again, the idea behind this index is that the better the robot assists the operator the lower is the muscle activation underlying the joint torque generation. Note that, with the same force exerted on the robot by the operator, the muscular effort may be different because of the upper-limb kinematics and dynamics strongly affects the efficiency of muscular contractions in generating joint toques (and consequently applied forces). The muscular activity is measured indirectly through surface electromyography, a technique to record the electrical activity produced by muscles during contraction; the stronger is the contraction, the higher is the electrical activity. Each EMG signal was elaborated as follows: (i) the DC offset was subtracted; (ii) the absolute value was calculated; (iii) the signal envelope was calculated using a Hilbert transform filter (Myers et al., 2003); (iv) the area under the EMG profile calculated (AEMG). For the purpose of this study, four upper-limb main muscles were considered to build the index. The index results from the sum of the four muscles area *AEMG*^*i*^:
  (8) $$\begin{array}{l} AEMG_{sum}=\overset{4}{\underset{i=1}{\sum }}AEMG^{i} \end{array}$$
  Finally, to compare movements of different lengths, the index was divided by Δ*x*_*r, z*_, the difference in height between the start and end points, yielding^1^:
  (9) $$\begin{array}{l} iEMG=\frac{AEMG_{sum}}{\Delta x_{r,z}} \end{array}$$
  where *AEMG*^*i*^ is the area underneath the envelope of the EMG of muscle *i*.

## 4. Control Parameters Tuning Procedure

### 4.1. Fuzzy Controller Implementation

In this section, the implementation of the fuzzy controller is shown, detailing the implemented parameters of the controller and the related motivations for their selection.

#### 4.1.1. Velocity Ranges Implementation

The following values have been imposed to the variables defined in (4) and in section 3.3: $v_{1}^{1}=0$ m/s, $v_{1}^{2}=0.01$ m/s, $v_{2}^{1}=0.005$ m/s, $v_{2}^{2}=0.025$ m/s, $v_{3}^{1}=0.02$ m/s, $v_{3}^{2}=0.04$ m/s, $v_{4}^{1}=0.04$ m/s, $v_{4}^{2}=\infty$ m/s, $v_{st}^{1}=0.04$ m/s, $v_{st}^{2}=0.06$ m/s. It has to be note that the velocity defining the upper bound of the safety transition range (i.e., $v_{st}^{2}$) has been defined to ensure safety while human and robot collaborate. In particular, such value has been calculated on the basis of the ISO 10218-1:2011, that limits the maximum collision force with the human to 65 N (for collision with the human face, i.e., the most critical scenario). Considering the impact formulation (Roveda et al., 2016) for a one degree of freedom in the Cartesian space:

(10) $$\begin{array}{l} -\left(1+\lambda \right)M_{r}{ẋ}_{r}=f_{max}\Delta t \end{array}$$

where λ is the coefficient of restitution of the impact (0 ≤ λ ≤ 1), the maximum robot impact velocity ẋ_*r*_ can be calculated in order to do not exceed the maximum force *f*_*max*_ = 65*N* in an impact time Δ*t*. Considering an impact time Δ*t* = 0.05 s (Haddadin et al., 2008), a robot mass *M*_*r*_ = 27 kg (i.e., the KUKA iiwa 14 R820 robot mass in motion—15 kg—plus the maximum robot payload—14 kg) and λ = 1 (to consider the worst impact condition), the maximum impact robot velocity results in $v_{st}^{2}=x_{r}=0.06$ m/s.

**Remark 8**. It has to be note that the velocity ranges have been *ad hoc* specified for the robot used in the experimental tests (i.e., the KUKA iiwa 14 R820 manipulator). The method is however general, allowing to specify different velocity ranges on the basis of the target manipulator, payload and application.

**Remark 9**. The implemented values are based on the experimental tests performed in Roveda et al. (2017b).

#### 4.1.2. Force and Derivative of the Force Input Membership Functions Implementation

The four sets of the force and derivative of the force input membership functions have been updated considering the four velocity ranges in (4). The values for $f_{i}^{j}$ and $df_{i}^{j}$ (where *i* indicates the considered velocity range and *j* the parameter index) have been implemented on the basis of the previous experimentation carried out in Roveda et al. (2017b). In particular: $f_{1}^{1}=110\left[N\right]$, $f_{1}^{2}=130\left[N\right]$, $df_{1}^{1}=30\left[N/s\right]$, $df_{1}^{2}=40\left[N/s\right]$,$f_{2}^{1}=100\left[N\right]$, $f_{2}^{2}=120\left[N\right]$, $df_{2}^{1}=20\left[N/s\right]$, $df_{2}^{2}=40\left[N/s\right]$,$f_{3}^{1}=90\left[N\right]$, $f_{3}^{2}=110\left[N\right]$, $df_{3}^{1}=15\left[N/s\right]$, $df_{3}^{2}=35\left[N/s\right]$,$f_{4}^{1}=65\left[N\right]$, $f_{4}^{2}=90\left[N\right]$, $df_{4}^{1}=10\left[N/s\right]$, $df_{4}^{2}=20\left[N/s\right]$.

In order to avoid any discontinuity while selecting the target membership function (both for the force and derivative of the force sets), the four velocity ranges have an overlap as defined in section 4.1.1. The assistance level *AL*^*i*^(*j, j*) is calculated for each set of input membership functions (with *i* identifying the corresponding set of input membership functions and *j* the degree of freedom). The assistance level *AL*(*j, j*) to be applied for the calculation of the impedance control set-point is then calculated as follows:

(11) $$\begin{array}{l} AL\left(j,j\right)=\sum AL^{i}\left(j,j\right)sf^{i}\left(j,j\right) \end{array}$$

where *sf*(*j, j*)^*i*^ is the shaping factor related to the ith set of input membership functions that are defined as follows:

(12) $$\{\begin{array}{ll} \; & \#1\;sf^{i}(j,j)=\frac{1+\sin\left(2\pi \frac{|{\dot{x}}_{r}(j)|-{\dot{x}}_{low}^{i}}{{\dot{x}}_{up}^{i}-{\dot{x}}_{low}^{i}}+{\dot{x}}_{low}^{i}+\frac{{\dot{x}}_{middle}^{i}-{\dot{x}}_{low}^{i}}{2}\right)}{2}\\ \; & \#2\;sf^{i}(j,j)=0 \end{array}$$

where ${ẋ}_{low}^{i}$, ${ẋ}_{up}^{i}$, and ${ẋ}_{middle}^{i}$ define respectively the lower limit, the upper limit and the central value of the velocity range *i* as detailed in (4). #1 is applied $if{ẋ}_{low}^{i}\leq |{ẋ}_{r}\left(j\right)|\leq {ẋ}_{up}^{i}$, while #2 is applied $if|{ẋ}_{r}\left(j\right)|<{ẋ}_{low}^{i}\;||\;|{ẋ}_{r}\left(j\right)|>{ẋ}_{up}^{i}$. On the basis of the above definition, *sf*^*i*^(*j, j*) is bounded in the range [0, 1]. In particular, if *sf*^*k*^(*j, j*) = 1, all the other *sf*^*i*^(*j, j*) = 0 (with *i* ≠ *k*), giving the only contribution to the definition of *AL*(*j, j*).

**Remark 10**. It has to be note that the parameter $f_{4}^{1}=65N$ has been imposed on the basis of the ISO 10218-1:2011. In fact, since the velocity range #4 considers the most critical situation (i.e., the highest velocity range) it is necessary to limit the interaction force at the level provided by the regulation.

**Remark 11**. For velocity range #4 (*i.e*., *i* = 4) ${ẋ}_{middle}^{i}=0.06$ m/s, ${ẋ}_{up}^{i}=0.08$ m/s. Moreover, *sf*^*i*^(*j, j*) = 1 if $|{ẋ}_{r}\left(j\right)|>{ẋ}_{up}^{i}$.

### 4.2. Neural Network Training Procedure

In this Section the Neural Network procedure to tune the control parameters is described.

#### 4.2.1. Participants

One of the author (i.e., Shaghayegh Haghshenas, female, 28 years old) performed all the Neural Network training experiments in order to have a consistent database from which to calculate the performance indexes related to the human-robot cooperation.

#### 4.2.2. Materials

The following materials have been involved in the experimental tests for training the above proposed Neural Network:

- the robotic platform KUKA iiwa 14 820 has been used for all the experiments;
- the FreeEMG300 (BTS Bioengineering, Italy), a 4 channels wireless EMG acquisition system, has been used to acquire the upper-limb EMG activity of the subject performing the experiments;
- surface EMG electrodes (Ambu Blue Sensor SE-00-S/50) have been used to collect the surface EMG signals;
- in order to measure the interactions between the human and the robot, the robot torque sensors at joint level has been exploited;
- a handle has been used in order to establish the interaction between the human and the robot.

**Remark 12**. It has to be underlined that the proposed approach can be applied to industrial manipulators that are not equipped with joint torque sensors. In that case, it is possible to adopt a force/torque sensor at the robot end-effector or exploit interaction force estimation based on motor currents measurements as in De Luca et al. (2006); Wahrburg et al. (2014) and Wahrburg et al. (2017).

#### 4.2.3. Subjects Preparation

Prior to the experiment execution, the surface EMG electrodes have been placed according to the SENIAM project recommendations (Stegeman and Hermens, 1998), on the following muscles: (i) the triceps lateral head, (ii) the deltoid anterior head, (iii) deltoid medial head, and (iv) the biceps long head.

#### 4.2.4. Experimental Procedure

**Figure 3:**
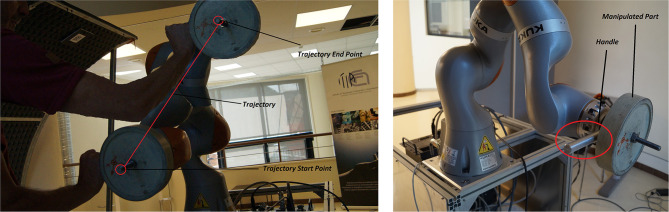
Experimental task and set-up. Robot-assisted part manipulation: task start and end positions along the task trajectory are shown. The 10 kg payload and the interaction handle (used by the human to interact with the robot) are shown.

The task consisted in a cooperative lifting of different weight-parts attached to the robot end-effector, following a trajectory involving all the three translational degrees of freedom while the rotational directions were constrained (Figure 3, showing task and set-up). On the basis of the inputs to the Neural Network described in section 3.2, the following values have been used for such parameters:

- values for the weight of the manipulated object *M*_*o*_: 0, 5, 10 kg
- values for the stiffness parameter of the impedance control *K*_*r*_: 1,000, 3,000, 5,000 N/m
- values for the damping ratio of the impedance control *h*_*r*_: 0.1, 0.25, 0.5, 0.75, 0.9
- values for *AL*_*med*_: *low, medium, high*
- values for *AL*_*med*_: *low, medium, high*

where terms *low, medium*, and *high* for each state of assistance level represent the numerical values (resulting in nine different fuzzy controller configurations) shown in Table 2.

**Table 2:** Assistance levels values applied for the training of the Neural Network.

**Assistance levels values for the NN training experiments**			
***AL*_*med*_, *AL*_*high*_**	**Low m**	**Medium m**	**high m**
*low* m	0.001, 0.0025	0.001, 0.0035	0.001, 0.0045
*medium* m	0.0025, 0.0045	0.0025, 0.0055	0.0025, 0.0065
*high* m	0.0035, 0.0065	0.0035, 0.0075	0.0035, 0.0085

The cooperative lifting test has been repeated (three times each) for all the possible combinations of input values imposed to the control algorithm, resulting in 405 experiments. For each experiment, the corresponding optimization indexes have been calculated as the outputs of the neural networks, mapping the interaction between the human and the robot.

**Remark 13**. The motivation behind the control parameters ranges definition in section 4.2.4 has to be note:

- mass range values: 0 kg corresponds to the absence of payload (to optimize the human-robot cooperation without any payload), 10 kg corresponds to the maximum payload that can be co-manipulated without safety limitation issues on motor torques (since the maximum robot payload is 14 kg and forces are also applied by the human to the robot, increasing the payload can result in exceeding motor torques limits);
- stiffness range values: 1,000 N/m has been considered as the lower bound for the optimization since too small values result in an extra deformation of the impedance control set-point ${\text{x}}_{r}^{0}$ with respect to the actual position **x**_*r*_. Since there is a limitation on $|\Delta {\text{x}}_{r}^{max}|=|{\text{x}}_{r}^{0}-{\text{x}}_{r}|<0.2$ m, exceeding such value result in safety stop of the robot. The upper bound has been imposed equal to the maximum stiffness value allowed by the robot impedance control;
- damping range values: in order to avoid a zero damping parameter (that may result in an unsafe and unstable robot behavior), the lower bound has been imposed equal to 0.1, while the upper bound has been imposed equal to the maximum damping value allowed by the robot impedance control;
- assistance levels ranges values: such values have been imposed on the basis of previous experimentation in Roveda et al. (2017b).

Since it is not possible to evaluate analytically the effect of each parameter on the defined rules (due the complex non-linear interaction dynamics) the optimization procedure have to consider the variation of the control parameters in order to achieve an optimized cooperation.

**Remark 14**. The proposed controller provides the assistance to the human operator by deforming the impedance control setpoint ${\text{x}}_{r}^{d}$. Therefore, the impedance control stiffness **K**_*r*_ cannot be imposed equal to zero. In such a case, in fact, the deformation of ${\text{x}}_{r}^{d}$ will not affect the human-robot interaction, without providing any assistance to the human. A minimum value of 500 N/m has to be used in order to provide assistance to the human.

### 4.3. Results

**Figure 4:**
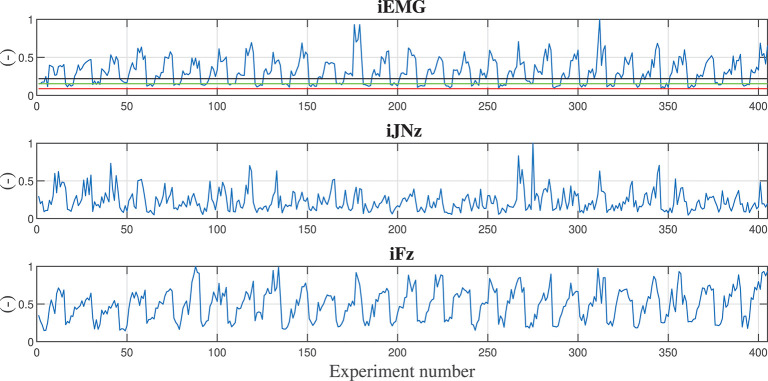
Calculated indexes during training experiments are shown along the *z* degree of freedom. First subplot shows the iEMG index evolution, second subplot shows the iNJz jerk index evolution along *z* direction, third subplot shows the iFz force index evolution along the *z* direction. In the first subplot, reference lines reporting the iEMG indexes relative to displacing the hand naturally (with no robot assistance) carrying 0.0, 1.5, and 3.0 kg weights are shown in red, green, and black, respectively. In order to have normalized and dimensionless indexes, each calculated value has been divided by the maximum obtained value in the training.

Considering the vertical *z* degree of freedom (that is the one in which the gravitational force is acting, i.e., the most critical degree of freedom while co-manipulating a heavy part), Figure 4 shows the calculated indexes (output data sets) for all the training experiments. The sequence of variation for the input parameters is: damping ratio, stiffness, mass of the manipulated component, assistance level *AL*_*high*_, assistance level *AL*_*med*_. Therefore, the first 5 values of the indexes calculation are related to the input parameters set in which only the damping ratio is varying: {*h*_*r*_, *K*_*r*_, *M*_*o*_, *AL*_*high*_, *AL*_*med*_} = {[0.1, 0.25, 0.5, 0.75, 0.9], 1, 000*N*/*m*, 0*kg, low, low*}. The first 15 values of the indexes calculation are related to the input parameters set in which the damping ratio and stiffness are varying: $\left\{h_{r},K_{r},M_{o},AL_{high},AL_{med}\right\}=\left\{\left[0.1,0.25,0.5,0.75,0.9\right],\left[1,3,5\right]10^{3}N/m,0kg,low,low\right\}$. The first 45 values of the indexes calculation are related to the input parameters set in which the damping ratio, stiffness, and manipulated object mass are varying: $\left\{h_{r},K_{r},M_{o},AL_{high},AL_{med}\right\}=\left\{\left[0.1,0.25,0.5,0.75,0.9\right],\left[1,3,5\right]10^{3}N/m,\left[0,5,10\right]kg,low,low\right\}$. From such analysis, it is clear that a repetitive trend can be identified on the basis of the parameters variation.

**Figure 5:**
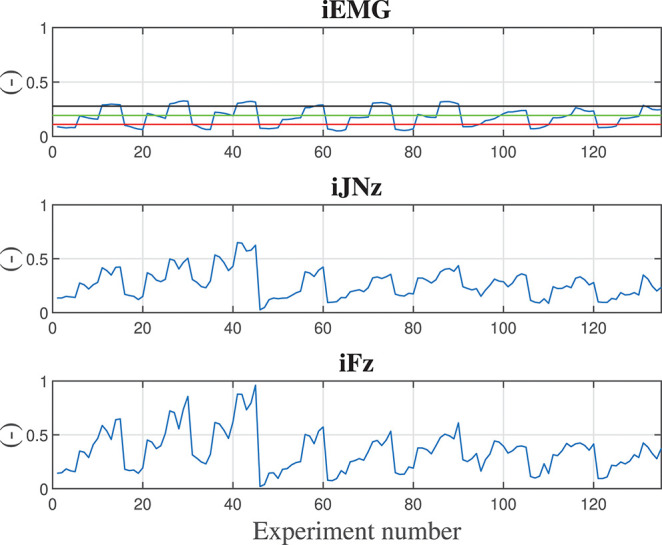
Calculated indexes considering a 10 kg component during training experiments are shown along the *z* degree of freedom. Subplot 1 shows the iEMG index evolution, subplot 2 shows the jerk index evolution along *z* direction, subplot 3 shows the force index evolution along the *z* direction. Reference lines reporting the iEMG indexes relative to displacing the hand naturally (with no robot assistance) carrying 0.0, 1.5, and 3.0 kg weights are shown in red, green, and black, respectively. In order to have normalized and dimensionless indexes, each calculated value has been divided by the maximum obtained value in the training.

In the first subplot of Figure 4, the iEMG index evolution is shown. Such index gives an indication of the muscular activation of the human during the cooperation and by varying the control parameters. As a baseline for comparison, the muscle activities of the human displacing the hand naturally (i.e., nor with robot assistance or presence of a weight, red line), lifting a 1.5 kg part (green line), and lifting a 3 kg part (black line) from the start point to the end point like the ones in the training experiments with the robot, are shown. In particular, it is shown that the proposed controller, also while manipulating the 10 kg part with weight unknown to the robot controller (as it can be further verified in Figure 5) reduces the muscular activity of the human, achieving better performance than in the case in which the human is manipulating a 1.5 kg part. Moreover, some control parameters sets are achieving a performance, which is equivalent to the one obtained during natural reaching without any weight.

In the second subplot of Figure 4, the jerk index evolution along the *z* direction is shown. Such index gives an indication of the smoothness of the movement of the human during the cooperation and by varying the control parameters.

In the third subplot of Figure 4, the force index evolution along the *z* direction is shown. Such index gives an indication of the effort in terms of applied force of the human during the cooperation and by varying the control parameters.

Neural Networks have been trained on the basis of the three defined indexes and on the basis of the Cartesian degree of freedom separately, with the input/output data sets. The standard network has been used for function fitting, meaning a two layer feed-forward network with a sigmoid transfer function in the hidden layer and a linear transfer function in the output layer. For each network, the optimal number of nodes in hidden layer, size of the network, has been chosen by doing a preliminary analysis on each network by evaluating the performance of the networks (via performance/regression plots) when varying hidden layer size in a wide range in MATLAB. After obtaining the desired training performance, trained networks have been fed with new input data sets significantly larger than the training data sets by considering all combinations of many possible values for damping ratio, stiffness, mass of the manipulated object, and assistance levels.

As a matter of example, Figure 5 shows the trained Neural Network for the considered indexes taking into account only the 10 kg manipulated component and the vertical *z* direction. From such training of the Neural Network it is possible to extract the control parameters optimizing the collaboration between the human and the robot. In particular, considering the three defined performance indexes, the optimized control parameters shown in Table 3 have been obtained. Looking at the impedance control parameters (i.e., stiffness and damping parameters), the stiffness parameter tends to be as small as possible, as expected to ensure a low-effort and smooth collaboration. The damping parameter adapts to the specific performance index: considering the smoothness index iJN, the damping ratio tends to saturate its value to smooth the collaboration trajectory; considering the force index iF, the damping ratio tends to minimize to avoid any opposition to the human motion that could result in increased interaction forces; considering the muscular activity index iEMG, the damping parameter adapts to minimize the muscular effort of the human operator. Considering the assistance levels *AL*_*med*_ and *AL*_*high*_, such parameters adapts in order to optimize the corresponding performance index.

**Table 3:** Optimized control parameters on the basis of the considered optimized performance index (OPI).

**Optimized control parameters for the 10 kg payload**				
**OPI**	***K*_*r, z*_*N*/*m***	***h*_*r, z*_**	***AL*_*med*_*m***	***AL*_*high*_*m***
iEMG	1,000	0.6	0.0025	0.0055
iNJz	1,000	0.1	0.001	0.006
iFz	1,000	0.95	0.0035	0.0035

**Figure 6:**
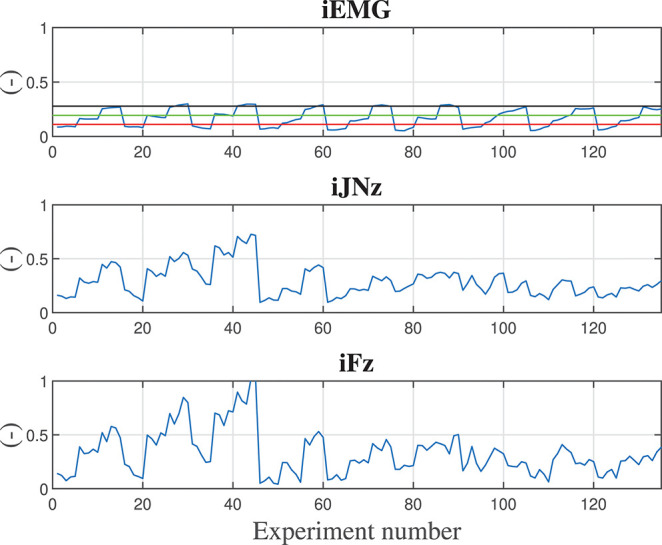
Calculated indexes considering a 5 kg component during training experiments are shown along the *z* degree of freedom. Subplot 1 shows the iEMG index evolution, subplot 2 shows the jerk index evolution along *z* direction, subplot 3 shows the force index evolution along the *z* direction. reference lines reporting the iEMG indexes relative to displacing the hand naturally (with no robot assistance) carrying 0.0, 1.5, and 3.0 kg weights are shown in red, green, and black, respectively. In order to have normalized and dimensionless indexes, each calculated value has been divided by the maximum obtained value in the training.

Considering, instead, a 5 kg manipulated component, Figure 6 shows the corresponding trained Neural Network. Table 4 shows the obtained optimized control parameters. The obtained parameters are slightly different with respect to the parameters obtained for the 10 kg payload but, however, are very close to each other. Therefore, on the basis of the on-line identification of the manipulated payload, a gains Table can be made from which to select the control parameters ensuring sub-optimal performance in the case of uncertainties related to the component weight estimation.

**Table 4:** Optimized control parameters on the basis of the considered optimized performance index (OPI).

**Optimized control parameters for the 5 kg payload**				
**OPI**	***K*_*r, z*_** ***N*/*m***	***h*_*r, z*_**	***AL*_*med*_** **m**	***AL*_*high*_** **m**
iEMG	1,000	0.5	0.0035	0.0055
iNJz	1,000	0.1	0.001	0.0055
iFz	1,000	0.95	0.001	0.0025

**Remark 15**. Considering the optimized control parameters from the trained network (refer for this to Tables 3, 4) uncertainties in the mass estimation result in sub-optimal collaboration performance. In fact, the optimized control parameters values rely on a limited set of values even in the presence of the manipulated mass variation, not dramatically decreasing the control performance if errors in the estimation of the payload are made. Therefore, the proposed approach can be applied in the cases of available estimation of the payload mass and in the case of unavailable estimation of the payload mass (initializing such value at the best knowledge of the operator executing the application).

### 4.4. ANOVA Analysis

In order to better understand the influence of the control parameters (i.e., *h*_*r*_, *K*_*r*_, *AL*_*high*_, *AL*_*med*_) and payload mass (i.e., *M*_*o*_) on the defined performance indexes in section 3.4.1 (i.e., *iNJ*, *iF*, and AEMG_*sum*_), the ANOVA analysis have been performed (Berens, 2009). Obtained results considering the vertical axis *z* (similar results are obtained for axis *x* and *y*) are shown in Table 5. The ANOVA analysis shows that the iEMG performance index is influenced by *K*_*r, z*_; the iNJz performance index is influenced by *K*_*r, z*_, *AL*_*med*_, *AL*_*high*_; the iFz performance index is influenced by all the considered parameters.

**Table 5:** *P*values defining the influence of control parameters and payload mass on the performance indexes are shown in the table.

**ANOVA results**					
	***P*_*value*_(*K*_*r, z*_)**	***P*_*value*_(*h*_*r, z*_)**	***P*_*value*_(*AL*_*med*_)**	***P*_*value*_(*AL*_*high*_)**	***P*_*value*_(*M*_*o*_)**
iEMG	0.	0.83	0.77	0.12	0.16
iNJz	0.	0.995	0.0023	0.0001	0.27
iFz	0.	0.0018	0.0036	0.	0.

## 5. Experimental Validation

Validation experiments have been performed implementing the proposed control algorithm to asses and compare the different control parameters sets obtained using the different optimization criteria. The implementation of such controllers don't take into account the weight of the manipulated part (i.e., the robot controller does not have any information about the part weight). Such controllers have been compared with the KUKA iiwa 14 R820 high-performance manual guidance controller having (i) the perfect compensation of the manipulated part weight (i.e., setting to the iiwa controller the exact manipulated payload), and (ii) a partial compensation of the manipulated part weight (i.e., an error equal to the 30% of the manipulated part weight has been introduced to the robot controller). In fact, in many real industrial cases, the weight of the part to be manipulated is not known accurately (as it is usually estimated from CAD software with errors ranging from 15 to 30%) and/or because the part to be manipulated next has a different weight (this is particularly true considering the Industry 4.0 paradigm, in which the robotic cell has to deal with a dynamic production). In such a way, the proposed approaches can be compared with an ideal high-performance control algorithm and with a more realistic industrial scenario. Indeed, five controllers have been tested and compared: (i) iEMG based optimized controller, (ii) iJN based optimized controller, (iii) iF based optimized controller, (iv) KUKA iiwa manual guidance controller with perfect compensation of the component weight, (v) KUKA iiwa manual guidance controller with uncertainties (30%) in the compensation of the component weight.

### 5.1. Participants

The following subjects have been involved in the evaluation of the proposed control approach:

- 20 healthy subjects (12 males, 8 females, with mean age = 29 ± 4 years) without any physical problem.

Prior to testing, all subjects have been informed about the evaluation scenario and the testing procedure.

### 5.2. Materials

The same materials described in section 4.2.2 have bee used for the validation of the proposed control approach.

### 5.3. Subjects Preparation

Each subject testing the proposed control approach has been prepared as described in section 4.2.3.

### 5.4. Experimental Procedure

The five different control algorithms have been implemented on the KUKA iiwa 14 R820 and a component of 10 kg weight has been attached to its end-effector, provided with a handle for the human-robot interaction. Human subjects are supposed to lift the component cooperatively with the robot up to a certain height (marked for the user awareness) for three times for each control algorithm. The trajectory performed by the human in interaction with the robot is online defined by the proposed controller on the basis of the interaction forces (i.e., no pre-defined trajectories are implemented). The human is, therefore, free to move the robot from the start point to the target endpoint. In total, each participant performs 15 lifting tasks to accurately evaluate the performance of each algorithm by filling out a provided questionnaire including the defined performance criteria. The running order of the control algorithms have been randomized to balance the evaluation results.

**Remark 16**. The control parameters implemented in this phase for the proposed controller are shown in Table 3. It has to be note that a gain-scheduling approach can be implemented, defining a gains Table from which to select on-line the control parameters on the basis of the estimated manipulated component weight. It has to be noted that the robot controller does not have any knowledge about the payload while executing the collaborative task.

#### 5.4.1. Qualitative Assessment

The performance of the different control methods and optimizations have been assessed using a questionnaire at the end of the evaluation experiments for all the 20 subjects. The following metrics have been defined to address both physical human-robot interactions (pHRI) and cognitive human-robot interactions (cHRI) to completely address human expectations and task goals (Rahman and Ikeura, 2016):

- *Naturalness*: human's overall likability, normalcy, ease of use, convenience, non- complexity in operation and collaboration.
- *Smoothness*: whether the movement is smooth.
- *Effort*: amount of effort, hardship or endeavor required to achieve a performance level satisfying the mental, physical and temporal demand.
- *Motion*: nature of object velocity and acceleration felt by the human (i.e., whether the velocity, acceleration is low or high compared to human expectation).
- *Stability*: presence/absence of oscillations, sudden inactivity of the system, and their effects on manipulation, object, system structure, and environment.
- *Detection of intention*: whether the robot follows human intention in accelerating or de-accelerating the motion.
- *Performance*: the overall performance, e.g. lifting the object to the desired position within the specified time and attempting to avoid user-unfriendly events.

**Figure 7:**
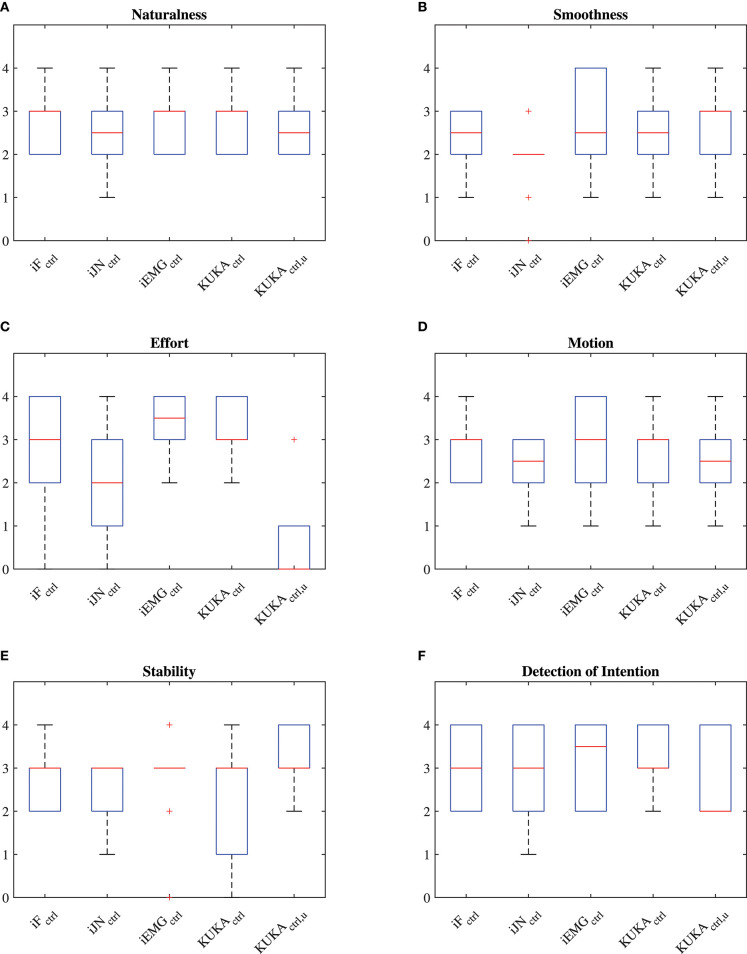
**(A)** Questionnaire results related to the *Naturalness* criteria of the questionnaire. **(B)** Questionnaire results related to the *Smoothness* criteria of the questionnaire. **(C)** Questionnaire results related to the *Effort* criteria of the questionnaire. **(D)** Questionnaire results related to the *Motion* criteria of the questionnaire. **(E)** Questionnaire results related to the *Stability* criteria of the questionnaire. **(F)** Questionnaire results related to the *Detection of Intention* criteria of the questionnaire.

**Figure 8:**
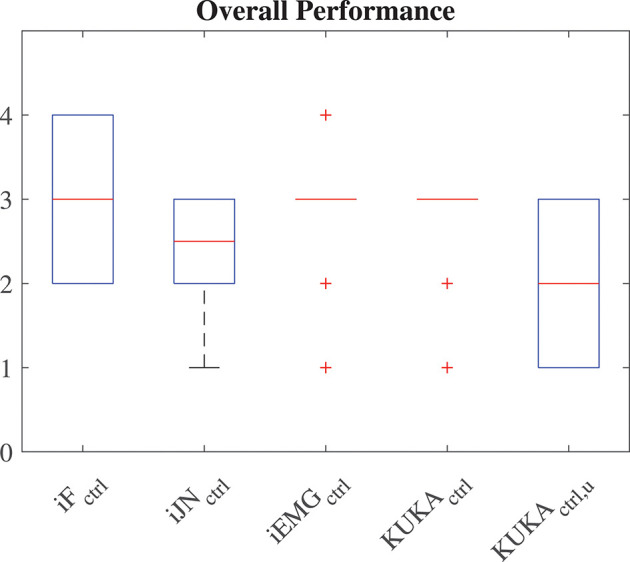
Questionnaire results related to the *Performance* criteria of the questionnaire.

The questionnaires scores of each performance criteria given by the 20 subjects to each controller are shown in Figure 7, and the overall performance in Figure 8 (considering the vertical *z* degree of freedom, results for the other DoFs are equivalent), in which the iF optimized controller is identified by *iF*_*ctrl*_, the iJN optimized controller is identified by *iJN*_*ctrl*_, the iEMG optimized controller is identified by *iEMG*_*ctrl*_, the KUKA iiwa manual guidance controller with perfect weight compensation is identified by *KUKA*_*ctrl*_, the KUKA iiwa manual guidance controller with uncertainties in the weight compensation is identified by *KUKA*_*ctrl, u*_. Results indicate that the fuzzy impedance controller optimized by the iEMG index is the most performant control algorithm. Such a controller is performing even better than the KUKA iiwa manual guidance controller with perfect compensation of the part weight. Further, it is important to underline that the *Effort* questionnaire criteria results (giving the load actually perceived by the subject while cooperating with the robot). Considering Figure 7, in fact, it is shown that even with an uncertainty of 30% of the total weight, the KUKA iiwa high-performance manual guidance controller becomes too heavy for the human-robot cooperation. Moreover, given the active assistance of the proposed controller, the developed approach performs better than the KUKA iiwa high-performance manual guidance controller with perfect part weight compensation.

#### 5.4.2. Quantitative Assessment

##### 5.4.2.1. Embedded safety rules evaluation

**Figure 9:**
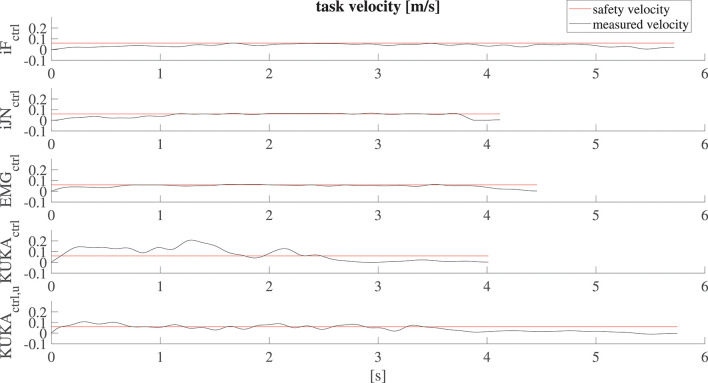
Results related to the robot velocity evaluation for subject 1 along the vertical direction *z*. The safety velocity limit is shown, highlighting that the proposed controller allows satisfying the defined limitation while the standard controller exceeds such limitation.

The defined embedded safety rules aiming to ensure safety in cooperative human-robot tasks can be evaluated by analyzing the velocities resulting from the collaborative task execution. Figure 9 shows the resulting velocities along the vertical direction *z* while subject 1 (similar results are obtained for all the subjects) is collaborating with the robot in the lifting task. The velocity profiles resulting from the application of the five applied controllers are shown. In particular, the safety velocity $v_{st}^{2}$ is highlighted in the plot in order to evaluate the safety performance of the controllers. In particular, it is shown that the proposed controllers allow satisfying the limitation of the velocity while the human-robot cooperation is performed.

##### 5.4.2.2. Empowering controller evaluation

**Figure 10:**
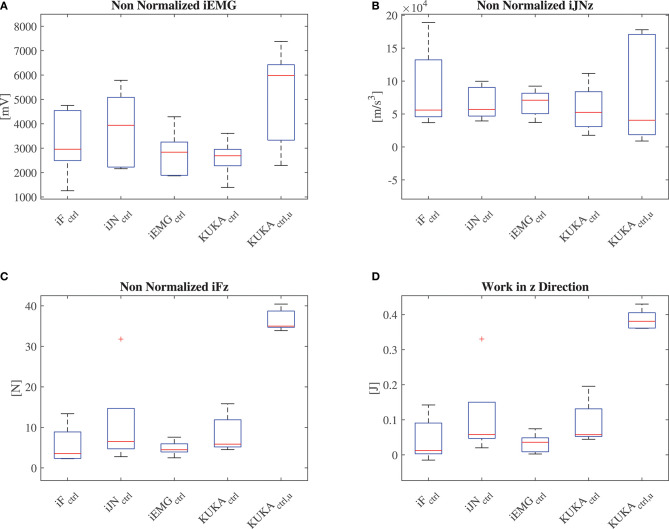
**(A)** Results related to the calculation of the (non-normalized) EMG index. **(B)** Results related to the calculation of the (non-normalized) position jerk index along the vertical direction *z*. **(C)** Results related to the calculation of the (non-normalized) force index along the vertical direction *z*. **(D)** Results related to the calculation of the work along the vertical direction *z*.

Besides the questionnaire based evaluation, the proposed performance indexes (iJN, iF, iEMG) without normalization, together with the total work resulting from the human-robot cooperation have been calculated for all the subjects during one lifting task along the vertical direction *z*. The results are shown in Figure 10. Considering the calculated indexes for the subjects, the fuzzy impedance controller optimized by the iEMG index is performing better than the other controllers. In fact, the related control parameters improved results with respect both the index *iEMG* and the index *iFz*. This demonstrates the effectiveness of the proposed optimization algorithm confirming the results related to the qualitative analysis.

**Remark 17**. It has to be note that, with respect to standard controllers, the proposed ones achieve lower interaction forces. Such a conclusion can be made on the basis of the results related to the effort index *iFz*.

##### 5.4.2.3. Installation task exploiting the proposed controller

**Figure 11:**
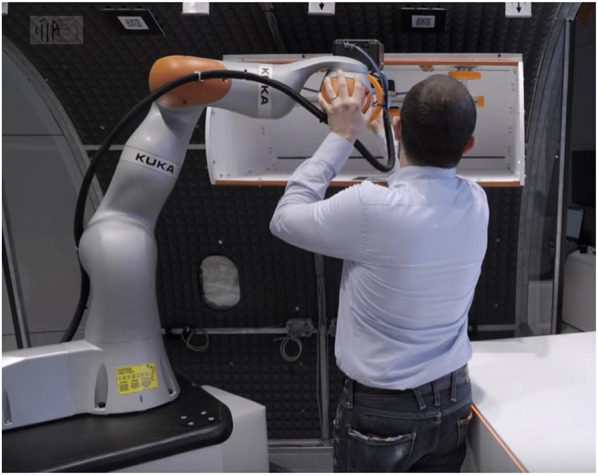
Installation task involving a bulky and heavy part exploiting the proposed controller.

The proposed controller has been applied to the installation task in https://www.youtube.com/watch?v=mdYpZZw93YE&t=17s (Figure 11). In particular, in order to assist the human operator in the installation of bulky and heavy components, the proposed controller allows to compensate for the part weight, while empowering the operator. A vision system allows to online define the main Cartesian path to be followed by the human during the task execution (by identifying parts positioning). The robot motion is then controlled on the basis of the human-robot interaction exploiting the proposed controller to track the defined Cartesian path.

## 6. Conclusions

The paper describes a cooperative fuzzy-impedance control with embedded safety rules to assist human operators in heavy industrial applications while manipulating unknown weight parts. On the basis of the described four main components the method is capable to provide the assistance to the human operator during the cooperation, relieving him/her from the manipulated unknown mass weight and ensuring safety at force and velocity levels. The capability of the controller to deal with unknown mass payload is important to improve the flexibility of operations inside the production plant (allowing to avoid any estimation procedure of the payload and high-precision *ad hoc* tuning of the control parameters and robot behaviors). In particular, in the case of high-variable production or highly customizable components (e.g., car bumpers), such a capability may result in saving a lot of set-up operations and, therefore, production time. However, the method is capable to include the estimation of the payload in the control algorithm, improving the cooperation.

Validation tests show, both with qualitative and quantitative tests, the improved achieved performance of the proposed controller, highlighting that the EMG based index optimization is the best performing algorithm. Safety is proven to be achieved with quantitative analysis. Moreover, even if the controller has been tuned on the basis of one single subject, results show that the approach is generalizable to other subjects.

This work presents some limitations that deserve attention: (i) the number of investigated muscles is limited to four and a deeper analysis involving other muscles is needed to verify whether the results are generalizable. (ii) the adoption of a on-line reinforcement learning approach instead of the adopted Neural Network approach can speed up the training phase of the proposed controller. Such approach is under development, allowing also the continuous learning of the control parameters during the daily cooperative tasks execution.

## Data Availability

The datasets generated for this study are available on request to the corresponding author.

## Ethics Statement

The paper only involves researchers from CNR, executing validation experiments of the HRC approach. Therefore, no permission has been asked for such standard industrial like experiments.

## Author Contributions

LR: methodology definition, protocol definition, approach implementation, and experiments. SH: experiments. MC: approach evaluation and experiments protocol verification. NP and LM: supervision.

### Conflict of Interest Statement

The authors declare that the research was conducted in the absence of any commercial or financial relationships that could be construed as a potential conflict of interest.

## Footnotes

^1^Since the Neural Network training experiments have been executed considering a reaching task as described in section 4.2.
